# *NEK1* variants and reduced protein levels in Chinese ALS patients: a descriptive study

**DOI:** 10.3389/fnagi.2026.1831861

**Published:** 2026-05-21

**Authors:** Xiaodan Chen, Yan Mo, Haishan Jiang

**Affiliations:** Nanfang Hospital, Southern Medical University, Guangzhou, Guangdong, China

**Keywords:** amyotrophic lateral sclerosis, clinical progression, genetic spectrum, *NEK1* variants, population differences, protein levels

## Abstract

**Objective:**

NIMA-related kinase 1 (*NEK1*) have been implicated in amyotrophic lateral sclerosis (ALS). But genetic spectrum and clinical presentation have not been systematically defined.

**Methods:**

We screened 378 ALS patients and identified *NEK1* variant carriers. Clinical records were reviewed retrospectively to characterise phenotypes. Leukocytes were isolated after routine testing and *NEK1* protein abundance was quantified by western blot to assess the relationship between *NEK1* protein levels and the rate of clinical progression. In parallel, we conducted a structured narrative review of published *NEK1*-ALS cases based on a systematic search of PubMed, Embase, and Web of Science. We extracted genetic and clinical information to summarise the variant spectrum, co-mutation profiles, and phenotype differences across populations.

**Results:**

*NEK1* variants were identified in 8 of 378 patients (2.12%). Protein analysis showed lower peripheral *NEK1* levels among carriers than among controls in this small exploratory sample. An exploratory analysis further suggested that lower systemic *NEK1* protein levels may be associated with faster disease progression; however, because these measurements were obtained from peripheral leukocytes at highly heterogeneous sampling times and without adjustment for major clinical confounders, they should be interpreted strictly as descriptive observations and do not support biomarker claims. While phenotypic heterogeneity and population-specific variant distributions were observed, these findings remain descriptive due to the small sample size.

**Conclusion:**

This regional case series provides a descriptive overview of *NEK1* variants in a Chinese ALS cohort and offers preliminary exploratory evidence consistent with reduced peripheral *NEK1* protein levels in variant carriers. The observed inverse relationship between lower measured protein levels and faster clinical decline should be regarded as hypothesis-generating only. Given the small sample size, highly heterogeneous sampling times, use of peripheral leukocytes, and lack of adjustment for major clinical confounders, these protein data do not support biomarker claims at this stage. Validation in larger, prospective multi-center cohorts with standardized longitudinal sampling is required.

## 1 Introduction

Amyotrophic lateral sclerosis (ALS) is a fatal neurodegenerative disorder characterised by progressive degeneration of upper and lower motor neurons (Boillée et al., 2006). It presents with steadily worsening muscle weakness and atrophy and ultimately leads to death, most commonly from respiratory failure (Tsai et al., 2017). The pathology of ALS involves multiple converging processes, with defective DNA damage repair and impaired proteostasis being particularly relevant to the role of *NEK1* (Ling et al., 2013; Sun et al., 2020). ALS is broadly classified into familial ALS (fALS) and sporadic ALS (sALS). Pathogenic variants can be identified in 90% of fALS and in 10% of sALS (Mathis et al., 2019). With the widespread use of high-throughput sequencing, more than 50 ALS-associated genes have now been confirmed (Fang et al., 2022). Expanding the ALS genetic landscape helps delineate clinical heterogeneity, providing a foundation for future studies to investigate potentially stratified management strategies (Suzuki et al., 2023; Xie et al., 2025).

NIMA-related kinase 1 (*NEK1*) was formally established in 2017 as one of the major susceptibility genes for ALS (Gratten et al., 2017). *NEK1* encodes a serine/threonine protein kinase implicated in key cellular processes, including cell-cycle regulation, the DNA damage response, ciliogenesis and mitochondrial homeostasis (Watanabe et al., 2025; Mann et al., 2023). Pathogenic *NEK1* variants are mainly associated with ALS and short-rib polydactyly syndrome type II (El Hokayem et al., 2012; Thiel et al., 2011), and loss-of-function (LoF) variants showing the strongest association with ALS risk, by contrast, missense variants are highly heterogeneous and their clinical significance remains uncertain, as many are also observed in the general population (Higelin et al., 2018; Naruse et al., 2021). Multiple cohorts and meta-analyses suggest that *NEK1* variants are detected in about 2–3% of ALS patients (Yao et al., 2021; Noh et al., 2025). To date, p.Arg261His is the missense variant most consistently supported as ALS-associated (Kenna et al., 2016). Functional studies further indicate that *NEK1* interacts with multiple ALS-related genes such as *C21ORF2* through shared signaling pathways (Watanabe et al., 2025; Gregorczyk et al., 2023), suggesting it may play an important regulatory role in the ALS disease network.

Despite growing clarity regarding the genetic relevance and potential molecular roles of *NEK1* in ALS, systematic characterization of the clinical phenotype associated with *NEK1* variants remains limited. Most studies have focused on variant frequency, pathogenicity classification, and risk estimation, whereas reports detailing age at onset, site of onset, progression rate, and overall phenotypic heterogeneity are comparatively sparse. It also remains unclear whether clinical features differ across populations. Despite its genetic relevance, the *in vivo* relationship between *NEK1* protein levels and disease progression remains unexplored. This study is designed as a descriptive case series supplemented by exploratory laboratory measurements to characterize *NEK1*-associated ALS in a Chinese cohort and investigate potential systemic protein correlates of disease trajectory. We acknowledge that the observed variability may stem from stochastic disease heterogeneity rather than a uniquely distinct genetic subtype.

## 2 Materials and methods

### 2.1 Study design and participants

All patients were evaluated according to the revised El Escorial criteria (Ludolph et al., 2015). This is a single-center descriptive case series. We retrospectively reviewed 378 patients with ALS who underwent genetic testing at Nanfang Hospital between January 2020 and December 2023 to identify *NEK1* variant carriers. Patients were classified as fALS if a definite ALS history was present in third-degree relative, those without a family history were classified as sALS. Early-onset ALS was defined as symptom onset before 40 years of age. The study was approved by the Ethics Committee of Nanfang Hospital (NFEC-2020-286), and all participants provided written informed consent prior to enrolment.

Demographic and clinical data were collected at admission, and the final follow-up was completed on 7 November 2025. Disease severity was assessed using the revised ALS Functional Rating Scale (ALSFRS-R) (Ortega-Hombrados et al., 2021). For the six patients included in the longitudinal ALSFRS-R analysis, the number of follow-up visits ranged from 4 to 6, with follow-up durations between 12 and 36 months and assessment intervals of approximately 3–6 months. The rate of disease progression during follow-up was calculated as the change in ALSFRS-R score over time between visits (points per month). Patients with an ALSFRS-R decline exceeding 0.5 points per month were classified as having rapid-progressing ALS (Gordon and Cheung, 2006; Tsai et al., 2020; Spisto et al., 2025; Nolano et al., 2024). Mood symptoms were assessed using the ALS-PHQ-9 Scale and the GAQ-7 Scale. Cognitive and behavioral status was reviewed retrospectively from routine clinical records; however, standardized ALS-specific neuropsychological classification according to contemporary ALS-FTD criteria was not systematically applied. Consequently, patients with subtle cognitive or behavioral changes could not be reliably classified as having mild cognitive impairment, mild behavioral impairment, combined mild cognitive-behavioral impairment, or overt ALS-FTD. Likewise, non-motor phenotyping was incomplete because formal autonomic testing and skin biopsy for small-fiber assessment were not performed. This lack of standardized cognitive and non-motor assessment limits the completeness of clinical phenotyping and reduces the strength of genotype–phenotype interpretation in the present cohort. Comprehensive electrophysiological phenotyping and transcranial magnetic stimulation (TMS) were also not performed in this cohort; therefore, potential differences in lower motor neuron burden, corticospinal involvement, and cortical excitability could not be systematically assessed.

### 2.2 Genetic analysis

Genomic DNA was extracted from peripheral blood using an automated 96-channel nucleic acid purification platform (CWE9600) together with a commercial blood DNA kit (CWE2100 Blood DNA Kit V2, CW2553). Sequencing libraries were prepared with the KAPA LTP Library Preparation Kit in accordance with the manufacturer’s protocol. After construction, libraries were vacuum-concentrated and underwent target capture via hybridization using the SureSelect enrichment system (Agilent Technologies). The captured libraries were subjected to Whole Exome Sequencing (WES) on the Illumina NovaSeq 6,000 platform. We ensured a mean coverage depth of >100×, with at least 95% of target bases covered at ≥20×. Raw data were processed using the GATK Best Practices pipeline (v4.2) for variant calling. All identified *NEK1* variants were validated by Sanger sequencing to ensure the robustness of our genetic findings.

Variants were filtered based on a minor allele frequency (MAF) < 0.1% in public databases, including gnomAD, ExAC, and 1,000 Genomes. Pathogenicity prediction was performed using in silico tools such as SIFT, PolyPhen-2, and CADD (score > 15), but these predictions were not used as stand-alone evidence of pathogenicity. Clinical significance was classified according to the ACMG/AMP 2015 guidelines as Pathogenic (P), Likely Pathogenic (LP), Variant of Uncertain Significance (VUS), or Likely Benign (LB), and VUS were interpreted separately from LP/P variants throughout the study. For the exploratory rare-variant burden analysis, we compared the frequency of rare *NEK1* variants in our cohort with ancestry-matched East Asian controls from the gnomAD database. Only variants with MAF < 0.1% in gnomAD EAS were included, and the comparison was restricted to coding and canonical splice-region variants captured by our exome design. Because our cases were sequenced on the Illumina NovaSeq 6,000 platform with a mean depth >100 × and ≥95% of target bases covered at ≥20×, whereas gnomAD EAS aggregates data from multiple sequencing platforms and pipelines, this comparison should be regarded as approximate rather than perfectly platform-matched. No formal principal-component-based correction for population structure was performed because only summary-level external-control data were available; instead, we minimized ancestry mismatch by restricting the comparison to the gnomAD East Asian subset. Accordingly, the burden analysis should be interpreted as exploratory and supportive rather than as a fully adjusted association test.

### 2.3 Leukocytes collected and western blot

While *NEK1* is ubiquitously expressed, protein levels measured in peripheral leukocytes were analyzed here only as an exploratory systemic readout and may not reflect *NEK1* biology in motor neurons or other disease-relevant tissues in ALS. In addition, blood sampling was performed at markedly heterogeneous time points across patients (3–90 months after symptom onset), and potential confounders including disease stage, treatment exposure, nutritional status, respiratory status, and intercurrent systemic conditions were not adjusted for. Accordingly, the following protein analysis should be interpreted as exploratory and hypothesis-generating only, and these data should not be used to support biomarker claims. Peripheral venous blood was collected in EDTA anticoagulant tubes and processed within 2 h. Leukocytes were isolated by percoll density gradient centrifugation (Cat#P8370, Solarbio), followed by centrifugation at 3,000 rpm for 5 min and washing with cold PBS. The leukocyte was resuspended in RIPA lysis buffer (P0013B, Beyotime) supplemented with protease inhibitors (P1005, Beyotime) and incubated on ice. After clarification by centrifugation, total protein concentration was determined using a BCA assay (BL521A, Biosharp). Equal amounts of total protein (20 μg) were loaded per lane; lanes were systematically arranged and explicitly labeled to facilitate direct visual comparison between individual patients (P1–P8) and healthy controls (C1–C3). Membranes were incubated with secondary antibodies and visualized using ECL. Densitometric analysis was performed using Image J, with *NEK1* levels normalized to the housekeeping protein *β*-actin. To ensure rigor, protein abundance for each patient was expressed as a numerical ratio relative to the mean of healthy controls, moving beyond purely qualitative assessment.

### 2.4 Structured narrative review of the literature

A structured literature search was conducted across PubMed, Embase, and Web of Science from database inception to October 31, 2025, to identify published reports of ALS cases carrying *NEK1* variants. The search strategy combined controlled vocabulary and free-text terms related to ALS and *NEK1*, including: (“amyotrophic lateral sclerosis” OR “motor neuron disease” OR ALS OR MND) AND (*NEK1* OR “NIMA-related kinase 1”). Reference lists of included articles were also screened manually to identify additional eligible studies.

Studies were included if they: (1) reported patients diagnosed with ALS or ALS-spectrum motor neuron disease; (2) provided individual-level or cohort-level data on *NEK1* variants; and (3) contained extractable information on at least one of the following: variant type, age at onset, site of onset, survival, cognitive/behavioral features, or co-mutations. Studies were excluded if they: (1) were review articles, conference abstracts without sufficient primary data, animal studies, or purely functional studies without clinical cases; (2) did not provide extractable clinical or genetic information; or (3) represented overlapping cohorts, in which case only the most comprehensive or most recent report was retained after cross-checking author lists, clinical centers, and recruitment periods.

The study selection process is summarized in Supplementary Figure S1. A total of 512 records were identified through database searching (PubMed, *n* = 146; Embase, *n* = 221; Web of Science, *n* = 145), and 11 additional records were identified through manual screening of reference lists. After removal of 148 duplicates, 375 records remained for title and abstract screening. Of these, 312 records were excluded as clearly irrelevant. The full texts of 63 articles were assessed for eligibility, of which 32 were excluded for the following reasons: lack of extractable *NEK1*-specific clinical/genetic data (*n* = 12), review articles or conference abstracts without primary data (*n* = 10), overlapping cohorts (*n* = 5), and non-human or purely functional studies without clinical cases (*n* = 5). Ultimately, 31 referenced studies were included in the structured narrative review (Gratten et al., 2017; Naruse et al., 2021; Tsai et al., 2020; Jiang et al., 2023; Pensato et al., 2025; Sánchez-Tejerina et al., 2022; Riva et al., 2022; Ma et al., 2022; Forsberg et al., 2019; Black et al., 2017; Yilmaz et al., 2022; Shu et al., 2018; Nel et al., 2022; McCann et al., 2020; Müller et al., 2018; Chen et al., 2022; Brenner et al., 2016; Lattante et al., 2021; Tripolszki et al., 2019; Liu et al., 2019; Liu et al., 2021; Nguyen et al., 2018; Zhang et al., 2020; Bartoletti-Stella et al., 2021; Libonati et al., 2024; Olsen et al., 2024; Leighton et al., 2024; Santangelo et al., 2024; Rifai et al., 2025; Ramos et al., 2019; Yang et al., 2025).

Two independent reviewers screened titles, abstracts, and full texts. Data extracted from eligible studies included publication year, country or ethnicity, number of *NEK1*-associated ALS cases, variant category, site of onset, age at onset, survival, cognitive involvement, and reported co-mutations. When available, the co-mutation profile was further categorized by gene, including *C9orf72*. Study quality was assessed using the Newcastle-Ottawa Scale (NOS) for observational studies when applicable. To improve transparency and reproducibility, a summary table of the referenced studies included in the current narrative synthesis is provided as Supplementary Table S1, listing the study design, country/ethnicity, number of *NEK1*-associated ALS cases, main variant categories, and the availability of key phenotypic variables.

Because of substantial heterogeneity in study design, case definition, reporting format, and completeness of individual clinical data, no formal meta-analysis or quantitative pooled effect estimation was performed. Therefore, this literature component should be interpreted as a structured narrative review based on a systematic search, rather than a formal systematic review with PRISMA-guided quantitative synthesis.

### 2.5 Statistical analysis

All statistical analyses were conducted using SPSS 26. Given the very small number of *NEK1* variant carriers (*n* = 8), this study should be regarded primarily as a descriptive case series, and all between-group comparisons are exploratory. Categorical variables were summarized as counts and percentages, and continuous variables as mean ± SD or median (range), as appropriate. Fisher’s exact test was used for exploratory comparisons involving categorical variables because of small cell counts, but *p*-values are presented for descriptive transparency only and should not be interpreted as evidence of formal statistical significance. For the exploratory rare-variant burden analysis, aggregated rare-variant counts in our cohort were descriptively compared with those reported in the gnomAD East Asian (EAS) reference dataset. Detailed odds ratios, nominal *p*-values, and confidence intervals are provided. Because the control dataset was external and summary-based, no individual-level adjustment for ancestry principal components, sequencing batch, platform, or coverage differences was possible. Therefore, this analysis should be interpreted only as an exploratory indication of possible enrichment, rather than as a precise estimate of population-level genetic risk or a formal association test. Image analysis and plotting were performed using Image J, GraphPad Prism 9.1, and Adobe Illustrator CC2021.

## 3 Results

### 3.1 Genetic analyses of *NEK1* variants in ALS patients

Between January 2020 and December 2023, 394 patients were diagnosed or suspected to have ALS, including 243 men and 151 women, and 8 patients reported a family history of ALS. All individuals with suspected ALS were advised to undergo whole-genome sequencing or next-generation sequencing to identify potential disease-associated variants. Among the 378 patients who completed genetic testing, the four most frequently implicated genes were *SETX*, *ANXA11*, and *NEK1*. Clinical characteristics of the overall cohort are summarized in Table 1.

**Table 1:** Demographic characteristics of ALS patients.

Variable	Subgroup/Unit	Value (*n*, % or Mean ± SD)
Sex	Male	243 (61.83%)
	Female	151 (38.17%)
Age at onset (years)	Mean ± SD	52.24 ± 12.47
	Early-onset (<40 years)	4 (1.05%)
Site of onset	Spinal (limb) onset	286 (72.77%)
	Upper limb onset	29 (7.38%)
	Lower limb onset	5 (1.27%)
	Bulbar onset	75 (19.08%)
	Mixed (Spinal+Bulbar) onset	32 (8.14%)
Family history	Familial ALS (fALS)	8 (2.04%)
	Sporadic ALS (sALS)	385 (97.96%)
Cognitive impairment	Yes	1 (0.25%)
	No	392 (99.75%)
ALSFRS-R score	Mean ± SD	36.73 ± 8.07
ALS disease progression rate	Rapid (>0.5 points/month)	3 (0.76%)
	Slow (≤0.5 points/month)	5 (1.27%)
Depression score	ALS-PHQ-9 (Mean ± SD)	6.36 ± 6.04
Anxiety score	GAQ-7 (Mean ± SD)	4.41 ± 4.91
Neuropathic pain	ALS-ID Pain (Mean ± SD)	0.852 ± 1.02
*NEK1* variant carriers	Identified (among 378 sequenced patients)	8 (2.12%)

Values are reported as means ± SD or as counts (%) where appropriate.

fALS, familial ALS; sALS, sporadic ALS; ALSFRS-R, ALS function rating scale-revised; ALS-PHQ-9, depression scale; GAQ-7, anxiety scale; ALS-ID Pain, neuropathic pain scale.

In our cohort, 8 patients (2.12%) carried heterozygous *NEK1* variants. To explore whether rare *NEK1* variants might be relatively enriched in this series, we performed an exploratory gene-based burden analysis using ancestry-matched controls from the gnomAD East Asian (EAS) database (Supplementary Table S2). To improve comparability, only rare variants with MAF < 0.1% in gnomAD EAS and within coding or canonical splice-related regions covered by our exome panel were included. Under this descriptive framework, rare *NEK1* variants appeared to be more frequent in our cohort than in the external gnomAD EAS reference dataset. However, because the case data were generated using a single-center Illumina NovaSeq 6,000 workflow with mean coverage >100×, whereas gnomAD EAS is a heterogeneous external reference compiled from multiple platforms, capture designs, and processing pipelines, and because no individual-level ancestry correction could be performed, this observation should be interpreted cautiously as an exploratory signal only, rather than as a fully adjusted estimate of genetic effect in the Chinese ALS population.

**Figure 1:**
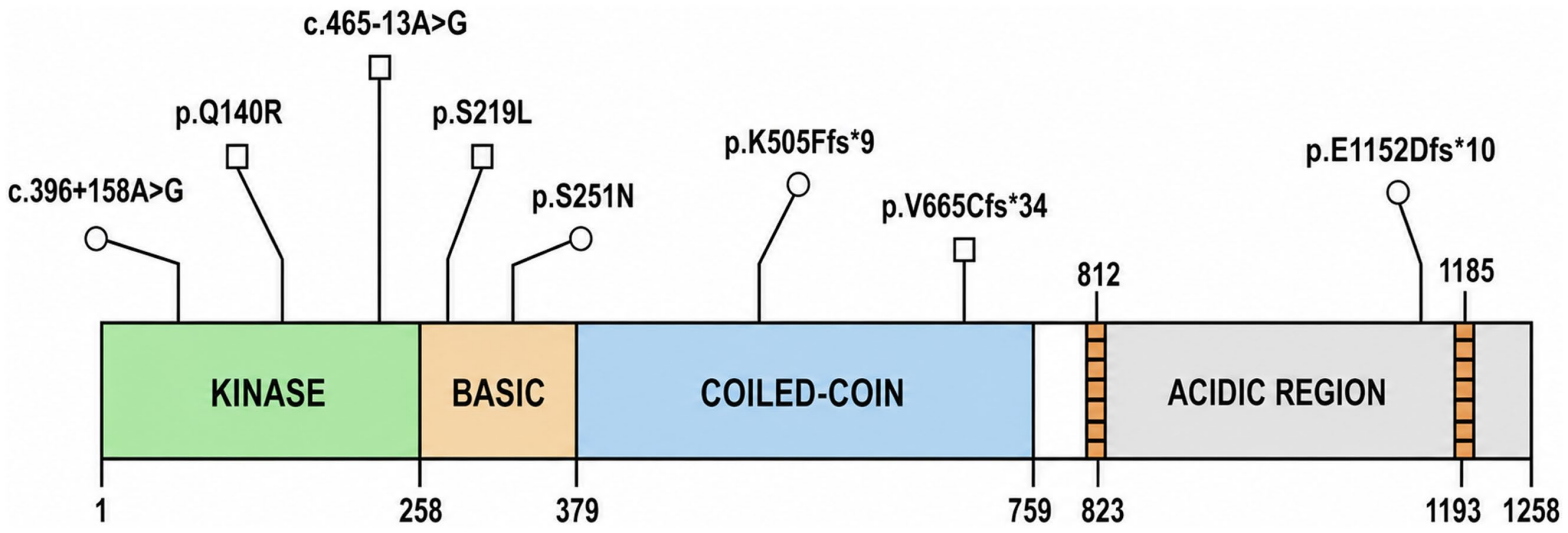
Schematic diagram of the *NEK1* protein structure. All the identified *NEK1* variants in our cohort are novel.

These included three frameshift deletion variants, two intronic variants, and three missense variants (Table 2). Based on ACMG/AMP criteria, the three frameshift deletion variants were classified as LP, four missense/intronic variants were classified as VUS, and c.396+158A>G was reassessed as LB because its population frequency exceeded our 0.1% filtering threshold. In the present study, only the LP variants were considered to have stronger evidence supporting disease relevance, whereas the VUS were retained for descriptive completeness only and were not interpreted as established disease-causing variants. We cross-referenced these variants with ClinVar to assess prior reporting status; however, novelty alone was not considered evidence of pathogenicity. Seven of the eight variants represented novel sites in our cohort, while c.396+158A>G was found to have a low frequency in the gnomAD database (MAF = 0.12% in East Asians); notably, we did not detect the recurrent variants p.Arg261His. The affected residues were located within highly conserved sequence regions across species (Supplementary Figure S2). Interestingly, the three likely pathogenic variants mapped to coiled-coil or intrinsically disordered regions, whereas the missense VUS clustered within the kinase domain (Figure 1). This positional observation is descriptive only and should not be interpreted as functional evidence of pathogenicity in the absence of direct experimental validation. To further annotate the two intronic variants (c.456-13A>G and c.396+158A>G), we performed in silico splicing analysis using SpliceAI and MaxEntScan. The c.456-13A>G variant was predicted to potentially affect the canonical acceptor site of exon 7 (SpliceAI score > 0.5); however, in the absence of transcript-level or functional validation, this result was considered supportive annotation only and was insufficient to upgrade the variant beyond VUS. In addition to *NEK1* variants, 2 patients also carried *C9orf72* hexanucleotide repeat expansions, defined in this study as pathogenic when the repeat number exceeded 30. No other ALS-associated variants were identified in the remaining 6 patients.

**Table 2:** List of the ALS patients carrying *NEK1* gene variants identified in our cohort.

Patient	Nucleotidic variants	Amminoacidic change	Exon	Hetero-or- homozygous	Pathogenicity (ACMG)	Additional variants
1	c.1992delA	p.V665Cfs*34	exon23	heterozygous	LP	NA
2	c.1513_1517del	p.K505Ffs*9	exon17	heterozygous	LP	NA
3	c.456-13A>G	/	Intron6	/	VUS	NA
4	c.656G>A	p.S219L	exon7	heterozygous	VUS	*C9orf72* repeat
5	c.3456delA	p.E1152Dfs*10	exon33	heterozygous	LP	*C9orf72* repeat
6	c.418G>A	p.G140R	exon7	heterozygous	VUS	NA
7	c.396+158A>G	/	Intron5	/	LB	NA
8	c.752C>T	p.S251N	exon9	heterozygous	VUS	NA

LP, likely pathogenic; VUS, variant of uncertain significance; LB, likely benign; NA, not available data.

*C9orf72* repeat expansion was considered pathogenic when the repeat number exceeded 30. VUS were included for descriptive completeness but were not interpreted as established disease-causing variants.

### 3.2 Demographics and clinical features of *NEK1* variants in ALS patients

The demographic and clinical characteristics of the 8 ALS patients carrying *NEK1* variants are summarized in Table 3. Because these variants differed in classification strength, clinical observations from carriers of LP variants and VUS were interpreted separately where possible. All 8 patients met diagnostic criteria for ALS. 4 patients developed ALS before the age of 45 years old, consistent with early-onset disease, and the remaining 4 showed a relatively narrow age-at-onset distribution, clustering between 50 and 60 years. By the last follow-up, 3 patients had died.

**Table 3:** Clinical and molecular features of ALS patients with *NEK1* variants after integration of sampling time and NEK1 protein levels.

Pt	Sex/Age at onset	Onset site	Variant type	Co-mutation	Sampling time (mo)	NEK1 protein level (Ratio)	ALSFRS-R progression (pts/mo)
1	M/55	Bulbar	LoF	None	14	0.15	0.36
2	M/44	Upper limb	LoF	*C9orf72*	90	0.1	0.22
3	M/40	Upper limb	Missense	None	7	0.23	0.48
4	M/59	Upper limb	Missense	None	3	0.35	0.64
5	M/43	Bulbar	LoF	*C9orf72*	18	0.05	0.67
6	F/55	Lower limb	Missense	None	12	0.18	0.41
7	M/43	Bulbar	Missense	None	10	0.2	0.52
8	F/59	Lower limb	Missense	None	5	0.3	0.33

NEK1 protein level is expressed as a ratio normalized to the mean of healthy controls (1.27 ± 0.13). ALSFRS-R progression rate was calculated as the change in ALSFRS-R score between serial follow-up visits divided by the follow-up interval (points/month). Longitudinal ALSFRS-R data were available for all eight patients, with 4–6 follow-up visits per patient over 12–36 months and assessment intervals of approximately 3–6 months.

Previous reports have suggested that hand-onset is a characteristic feature of *NEK1*-associated ALS (Tsai et al., 2020). Among the three patients with hand-onset, disease trajectories varied significantly, ranging from slow progression with late-stage bulbar involvement (Patient 2) to rapid onset of bulbar symptoms within one year (Patient 4). 3 patients presented with predominant bulbar onset, and the remaining 2 patients had lower-limb onset. None of the 8 patients had a family history of ALS or showed clinically overt dementia during routine follow-up. However, because standardized ALS-specific cognitive and behavioral assessments were not systematically performed, mild cognitive impairment, mild behavioral impairment, or combined mild cognitive-behavioral impairment could not be reliably excluded. This distinction is important because recent work suggests that even subtle cognitive and behavioral changes in ALS may carry prognostic relevance (Spisto et al., 2025). Accordingly, the present dataset does not support fine-grained clinical subtyping or strong genotype–phenotype inferences regarding cognitive or non-motor involvement. We attempted segregation analysis in all available families; however, DNA samples from first-degree relatives were unavailable for most cases, which limited formal co-segregation assessment. In Patient 2, although the parents were deceased and could not be tested, targeted Sanger validation showed that his two asymptomatic adult children (aged 21 and 24 years at last contact) did not carry the p.K505Ffs9 variant. In Patient 5, one unaffected elder sibling who agreed to testing was also negative for the p.E1152Dfs*10 variant*. These observations provide limited family-based information but are insufficient to establish formal segregation because of the small number of informative relatives and the possibility of age-dependent penetrance. Given the very small number of carriers (*n* = 8), the following subgroup comparisons between LoF and missense variants are strictly descriptive (Table 4). The reported differences represent observed trends within this specific series and lack the statistical power to support generalized clinical conclusions.

**Table 4:** Comparison of clinical characteristics between ALS patients with and without *NEK1* variants.

Variable	*NEK1*^+^ (*n* = 8)	*NEK1*^−^ (*n* = 370)	Exploratory *p*-value (Fisher’s exact test)
Age at onset (years)	49.75 ± 7.74	52.40 ± 12.60	>0.05
Male sex, *n* (%)	6 (75.0%)	237 (64.1%)	>0.05
Early-onset ALS (<40 years), *n* (%)	4 (50.0%)	10 (2.7%)	<0.001*
Spinal onset, *n* (%)	5 (62.5%)	281 (75.9%)	>0.05
Bulbar onset, *n* (%)	3 (37.5%)	72 (19.5%)	>0.05
Mixed onset, *n* (%)	0	32 (8.6%)	–
Familial ALS, *n* (%)	0 (0%)	8 (2.2%)	>0.05
Dementia, *n* (%)	0	1 (0.3%)	–
ALSFRS-R score (baseline)	~36	36.73 ± 8.07	>0.05
ALSFRS-R progression >0.5/mo, *n* (%)	3 (37.5%)	~22 (5.9%)	<0.01*
ALS-PHQ-9 (depression), mean ± SD	6.6 ± 4.1^†^	6.36 ± 6.04	>0.05
GAQ-7 (anxiety), mean ± SD	~4.5	4.41 ± 4.91	>0.05
ALS-ID Pain, mean ± SD	~0.9	0.852 ± 1.02	>0.05
Deceased at follow-up, *n* (%)	3 (37.5%)	~63 (17%)	>0.05

**Figure 2:**
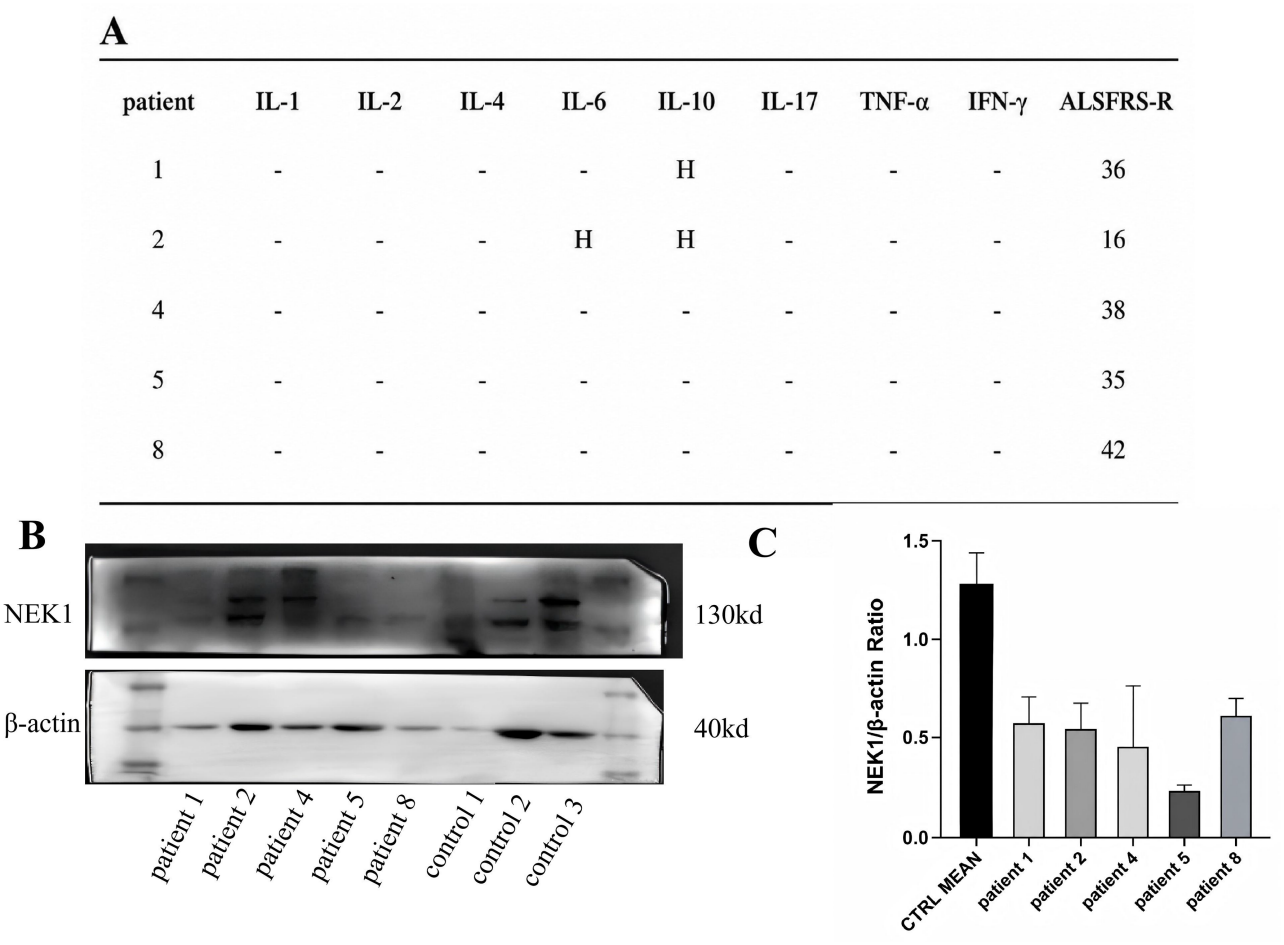
Systemic *NEK1* levels and inflammatory profiles. **(A)** Individual levels of inflammatory cytokines and baseline ALSFRS-R scores. **(B)** Representative Western blot image with specific lanes explicitly labeled for variant carriers and representative healthy controls to ensure clear identification. **(C)** Relative densitometric quantification of *NEK1* protein normalized to the *β*-actin loading control. Numerical values represent the *NEK1*/β-actin grey value ratio normalized against the healthy control mean (1.27 ± 0.13). Exploratory Pearson correlation analysis showed a descriptive inverse relationship between normalized *NEK1* protein levels and the rate of ALSFRS-R decline (*r* = −0.741, nominal *p* = 0.046, *n* = 5), which should be interpreted cautiously because of the very small sample size, highly heterogeneous sampling times, the use of peripheral leukocytes, and the absence of adjustment for major clinical confounders. These exploratory data do not support biomarker claims. H/↑ indicates elevated levels. Data are presented as mean ± SD.

*Nominal *p* < 0.05 in exploratory comparison; ^†^Estimated from Figure 2. Data are presented as mean ± SD or *n* (%). Given the very small number of *NEK1* carriers, all comparisons should be interpreted descriptively rather than inferentially.

LoF variants have been reported as an important genetic risk factor for ALS (Naruse et al., 2021); however, substantial phenotypic variability was observed even among the three patients carrying LoF variants in our cohort, and interpretation should be cautious because 2 of these 3 patients also harbored *C9orf72* repeat expansions. Among the 3 patients with loss-of-function variants, 2 patients had bulbar onset and showed a relatively rapid course, whereas the third patient presented with left hand onset and progressed more slowly, surviving for 10 years from symptom onset to death. Notably, the two rapidly progressive LoF carriers both had coexisting *C9orf72* repeat expansions; therefore, these clinical features cannot be attributed to *NEK1* LoF variants alone. The remaining 5 patients carried variants classified as VUS or LB and mostly had limb onset, with only one showing bulbar onset. Because these variants lack sufficient evidence for pathogenicity, their associated clinical features should not be interpreted as defining a *NEK1*-associated phenotype. Their disease progression also varied considerably, further emphasizing that phenotypic heterogeneity in this small descriptive series cannot be attributed solely to *NEK1*, particularly for variants that remain classified as VUS. Neuropathic pain scores were recorded descriptively in our cohort, but these findings were not analyzed further. This symptom should be interpreted within the broader context that ALS is increasingly recognized as a multisystem disorder with non-motor sensory involvement, rather than as a purely motor syndrome (Nolano et al., 2024). The most rapidly progressing patient underwent gastrostomy 22 months after symptom onset. By contrast, the slower-progressing patient had reached 3 years at the last follow-up with only mild upper-limb involvement. A descriptive comparison between patients carrying *NEK1* loss-of-function and non-LoF variants is shown in Table 5; however, these subgroup findings should not be interpreted as evidence of an independent phenotype–genotype effect of *NEK1* LoF variants because of the very small sample size and the presence of *C9orf72* co-mutations in 2 of the 3 LoF carriers.

**Table 5:** Comparison between ALS patients carrying *NEK1* loss-of-function and missense variants.

Variable	LoF (*n* = 3)	Missense (*n* = 5)	Exploratory *p*-value (Fisher’s exact test)
Age at onset (years)	47.3 ± 6.1	51.2 ± 8.2	>0.05
Male sex, *n* (%)	3 (100%)	3 (60%)	>0.05
Early-onset ALS (<45 yrs), *n* (%)	2 (66.7%)	2 (40%)	>0.05
Bulbar onset, *n* (%)	2 (66.7%)	1 (20%)	>0.05
Upper limb onset, *n* (%)	1 (33.3%)	3 (60%)	–
Lower limb onset, *n* (%)	0	2 (40%)	–
ALSFRS-R progression rate (pts/mo)	0.56 ± 0.16	0.48 ± 0.17	>0.05
Rapid progressors, *n* (%)	2 (66.7%)	1 (20%)	–
Survival time, months	~74.3 ± 22.0	~55.4 ± 18.7	>0.05
Death at follow-up, *n* (%)	1 (33.3%)	2 (40%)	–
Co-mutation with *C9orf72*, *n* (%)	2 (66.7%)	0 (0%)	<0.05*

*Nominal *p* < 0.05 in exploratory comparison; − Not applicable or not calculated. ALSFRS-R progression rate is summarized descriptively. Because the subgroup sample sizes were extremely small (LoF *n* = 3; non-LoF *n* = 5), these comparisons should not be interpreted as inferential evidence.

### 3.3 The relationship between *NEK1* protein level at peripheral system and disease progression in ALS patients

To explore whether peripheral *NEK1* protein levels might show a descriptive relationship with clinical progression in this small sample, we performed an exploratory semi-quantitative western blot analysis. Leukocyte inflammatory markers and ALSFRS-R scores were recorded at the time of sampling for contextual interpretation; however, these measurements were not sufficient to control for major clinical confounders. Therefore, this analysis was designed only to provide a descriptive systemic snapshot, rather than evidence for a clinically applicable biomarker.

Peripheral blood for *NEK1* protein analysis was collected at highly heterogeneous disease stages, with sampling times ranging from 3 to 90 months after symptom onset (Table 3; Figure 2A). This wide distribution is important for interpretation because protein abundance may vary with disease stage and other time-dependent clinical factors. Inflammatory cytokine profiling was largely unremarkable, with only minor elevations observed in two carriers (Figure 2A). Given the limited sample size and lack of longitudinal immune profiling, these inflammatory findings are presented descriptively and should not be overinterpreted.

To enhance interpretability, Western blot lanes were clearly labeled by patient and control groups, and all signals were normalized to the *β*-actin loading control (see Figure 2B). Nevertheless, whole-leukocyte lysates are not disease-relevant tissue for ALS pathogenesis, and the cross-sectional design together with heterogeneous sampling times precludes mechanistic, temporal, or causal inference. These pilot data should therefore be viewed strictly as descriptive exploratory observations and do not support the conclusion that systemic *NEK1* protein is a biomarker or that it directly reflects central nervous system pathology.

Normalized *NEK1* protein levels (*NEK1*/β-actin ratio) were lower in *NEK1* variant carriers than in the small healthy control group (Figure 2C). The lowest values were observed in the three loss-of-function (LoF) carriers (Patients 1, 2, and 5). However, because these measurements were obtained from peripheral leukocytes in a limited cross-sectional sample with markedly heterogeneous sampling times, they should be interpreted as preliminary descriptive observations only, rather than direct *in vivo* functional validation or evidence of biomarker performance.

In a hypothesis-generating analysis restricted to the patients with available longitudinal ALSFRS-R data, each patient had 4–6 follow-up visits over 12–36 months, with ALSFRS-R assessments approximately every 3–6 months. We observed a descriptive inverse correlation between normalized *NEK1* protein level and the rate of ALSFRS-R decline (Pearson r = −0.741, nominal *p* = 0.046). Because this analysis was based on a very small sample, used peripheral leukocyte samples collected at highly heterogeneous time points, and did not adjust for important clinical covariates such as disease stage, treatment exposure, nutritional status, respiratory status, or timing of sampling relative to disease onset, it should be interpreted strictly as exploratory and hypothesis-generating. These data are insufficient to support biomarker claims. Individual-level laboratory findings and clinical progression rates are integrated into Table 3.

### 3.4 An overview of ALS patients with *NEK1* variants

Our structured narrative review included 31 eligible studies comprising 287 previously reported *NEK1*-associated ALS cases. Across these studies, the global genetic landscape was dominated by missense variants (69%) over loss-of-function (LoF) variants (31%), consistent with a polygenic risk model (Gratten et al., 2017; Naruse et al., 2021; Tsai et al., 2020; Jiang et al., 2023; Pensato et al., 2025; Sánchez-Tejerina et al., 2022; Riva et al., 2022; Ma et al., 2022; Forsberg et al., 2019; Black et al., 2017; Yilmaz et al., 2022; Shu et al., 2018; Nel et al., 2022; McCann et al., 2020; Müller et al., 2018; Chen et al., 2022; Brenner et al., 2016; Lattante et al., 2021; Tripolszki et al., 2019; Liu et al., 2019; Liu et al., 2021; Nguyen et al., 2018; Zhang et al., 2020; Bartoletti-Stella et al., 2021; Libonati et al., 2024; Olsen et al., 2024; Leighton et al., 2024; Santangelo et al., 2024; Rifai et al., 2025; Ramos et al., 2019; Yang et al., 2025; Table 6).

**Table 6:** Clinical and genetic features of ALS patients with *NEK1* variants in previously reported studies.

Index	Asians	Caucasians	Others	Total	*p*
Sex, M/F	112, 63	49, 50	20, 10	159	0.876
Variant type, *n*					0.008
LoF	38	25	12	75	
Missense	121	80	30	231	
Additional variants, *n*	37	25	12	74	0.325
Site of onset, *n* (%)	102	80	40	222	0.072
Spinal	80 (78.4)	60 (75.0)	30 (75.0)	170 (76.5)	
Upper limb	40 (39.2)	30 (37.5)	10 (25.0)	80 (36.0)	
Lower limb	31 (30.4)	15 (18.8)	5 (12.5)	51 (23.0)	
Bulbar	22 (21.6)	25 (31.3)	10 (25.0)	57 (25.7)	
Age at onset, *n*, years	55.61 ± 11.23	59.52 ± 11.22	57.00 ± 9.56	58.10 ± 10.35	0.034
LoF	57.81 ± 9.95	58.28 ± 10.54	55.00 ± 8.45	57.35 ± 9.65	0.8
Missense	53.26 ± 12.19	59.89 ± 11.45	57.80 ± 10.12	56.80 ± 11.85	0.02
Survival time, months	38.91 ± 4.51	50.69 ± 4.57	47.00 ± 6.00	45.12 ± 5.42	0.119
LoF	40.58 ± 8.02	47.86 ± 6.93	42.00 ± 8.30	44.20 ± 7.53	0.417
Missense	37.89 ± 5.51	51.36 ± 5.44	45.00 ± 6.00	47.30 ± 5.70	0.184
Cognitive impairment, *n*	15	11	2	28	0.097

M, male; F, female; LoF, loss of function; SD, standard deviation; SE, standard error.

*p*, the comparison between Asians and Caucasians group.

Information on additional variants was not uniformly available across all included studies. Among the 74 cases with reported co-mutation data, 18 cases carried concomitant *C9orf72* repeat expansions.

Aggregating all previously reported ALS cohorts carrying *NEK1* variants, we did not observe clear descriptive differences in age at onset or survival between patients with LoF variants and those with missense variants. Among the 287 previously reported *NEK1*-associated ALS cases, information on additional genetic variants was available for 74 cases; of these, 18 were reported to carry concomitant *C9orf72* repeat expansions. However, because *C9orf72* testing or co-mutation reporting was not uniformly performed across studies, this number should be regarded as a minimum estimate rather than the true prevalence of *C9orf72* co-mutation among *NEK1*-ALS cases. We therefore further compared clinical characteristics across populations from different regions. There was no significant difference in sex distribution between Asians and Caucasians carrying *NEK1* variants. However, marked differences were observed in the spectrum of *NEK1* variant types across the two populations.

Cross-population analysis revealed distinct genetic and clinical signatures: Asian carriers harbor a higher proportion of LoF variants and exhibit a pronounced tendency toward upper-limb (particularly hand) onset compared to Caucasians, who show more frequent missense variants and a higher incidence of bulbar onset.

Our synthesis of previously reported *NEK1*-ALS cases revealed a trend toward earlier age at onset in Asian populations compared with Caucasians (Table 6). However, this observation should be interpreted cautiously, as it may reflect differences in healthcare-seeking behavior, age at referral, or variable sequencing practices across studies, rather than a genuine biological distinction. In contrast to age at onset, no significant difference in survival time was observed between Asian and Caucasian *NEK1* carriers. Survival duration was highly variable within both groups: among Asian patients, survival ranged from 8.8 to 120 months, while among Caucasians, the range extended from 7.5 to 230 months. Despite a numerically earlier age at onset and shorter mean survival in Asian cohorts, these differences were not statistically significant, suggesting that the clinical course of *NEK1*-ALS remains broadly comparable across ethnicities once adjusted for potential referral biases.

Unlike sALS caused by genes such as *SETX* (Chen et al., 2025), which typically has an early onset, *NEK1*-associated fALS tends to begin at an age broadly similar to *NEK1*-associated sALS, only 3 juvenile-onset cases have been reported with additional variants in the literature to date (Jiang et al., 2023; Sánchez-Tejerina et al., 2022). Geographic variations extend to family history, which is more frequently reported in European than Asian cohorts. Furthermore, the recurrent presence of co-mutations—most notably *C9orf72*—highlights *NEK1*’s role within an oligogenic framework that likely contributes to clinical heterogeneity. Nevertheless, interpretation should remain cautious because many published studies did not systematically report whether *C9orf72* testing was performed or whether additional variants were excluded, limiting our ability to determine the true frequency and phenotypic impact of *C9orf72* co-mutation in previously reported *NEK1*-ALS cases. Since these genes have been linked to DNA damage and genome repair pathways, the impact of having more than one disease-associated variant may go beyond a simple combination of symptoms and could reflect interactions between genes.

## 4 Discussion

In this study, we combined ALS cases carrying *NEK1* variants from our cohort with previously reported cases in the literature and provided a structured narrative synthesis of their variant spectrum and clinical phenotypes. However, because the evidentiary strength differed substantially across variants, particularly between LP variants and VUS, these categories should not be interpreted equivalently. A further point requiring caution is that several variants in our cohort remained classified as VUS. These variants were retained to illustrate the observed genetic diversity of *NEK1* in this cohort, but they should not be interpreted as proven ALS-causing mutations. In particular, in silico prediction, domain localization, and rarity in population databases are insufficient to establish pathogenicity without segregation evidence, transcript analysis, or functional validation. In total, we collated 302 unique *NEK1* variant sites, including 99 LoF variants and 203 missense variants. The most frequently reported variants were p.Ser1036 and p.Arg261His. In our cohort, the overall detection rate of *NEK1* variants was about 2.1%, slightly lower than that reported in other Chinese cohorts, with LoF variants accounting for 1.3% and missense variants for 0.8%. This distribution differs from prior reports suggesting that missense variants are more prevalent, which may be due to the relatively small sample in our study.

Overall, *NEK1* variants did not preferentially cluster at any specific sites across the gene. However, when our cohort was considered alongside prior reports, LoF variants appeared more likely to fall within regions of currently uncertain functional annotation, whereas missense variants did not show a comparable enrichment. However, when we considered our cohort alongside prior studies, loss of function variants appeared more likely to cluster in domains whose functions remain unclear, whereas missense variants did not show the same tendency. It is hypothesized that loss-of-function variants might contribute to disease primarily through haploinsufficiency, where a critical reduction in protein abundance potentially triggers neurodegeneration. Furthermore, uncharacterized domains could theoretically harbor regulatory sites, though experimental validation is necessary to confirm whether their disruption directly impairs *NEK1* function in ALS. If LoF variants occur in critical domains, they may be more likely to cause lethal skeletal ciliopathies such as short rib polydactyly syndrome, which would make these individuals unlikely to be included in ALS cohorts. Alternatively, missense variants might only exert a pathogenic effect when localized within essential domains that govern substrate recognition or enzymatic activity, a possibility that warrants further structural and functional investigation. Missense variants in uncharacterized domains may have effects that are too subtle to detect, which could explain why LoF variants are mainly found in these domains, whereas missense variants are enriched in domains with established functions.

In addition to *NEK1* variants, we identified 2 patients in our ALS cohort who carried a *C9orf72* hexanucleotide repeat expansion, which was defined as pathogenic when the repeat number exceeded 30 in this study. An increasing body of evidence suggests that ALS does not fully conform to the classic “gene-phenotype” model. In previously reported *NEK1*-related ALS cases, coexisting variants across multiple genes have also been observed. Besides *C9orf72*, variants have been reported together with changes in more than 20 other ALS associated genes, including *SOD1*, *FUS*, and *OPTN* (Gratten et al., 2017; Jiang et al., 2023; Pensato et al., 2025; Sánchez-Tejerina et al., 2022; Riva et al., 2022; Ma et al., 2022; Forsberg et al., 2019; Black et al., 2017; Yilmaz et al., 2022; Shu et al., 2018; Nel et al., 2022; McCann et al., 2020; Müller et al., 2018; Chen et al., 2022; Brenner et al., 2016; Lattante et al., 2021; Tripolszki et al., 2019; Liu et al., 2019; Liu et al., 2021; Nguyen et al., 2018; Zhang et al., 2020). Co-existing variants often contribute to clinical heterogeneity. Patients who also carry *FUS* variants tend to have an earlier onset or faster disease progression (Jiang et al., 2023; Pensato et al., 2025), whereas those with co-existing *C9orf72* variants may develop cognitive and behavioral involvement, including dementia (Lattante et al., 2021; Nguyen et al., 2018). Moreover, in some ALS patients carrying *NEK1* variants, *NEK1* may not be the primary disease driving gene. Instead, it may contribute to disease only in the presence of a particular genetic background or additional molecular events (Riva et al., 2022). Further analyses indicate that among ALS patients with two or more genetic alterations, *C9orf72* is the most common co-mutated gene. Therefore, in our cohort, the clinical manifestations observed in the two *NEK1* LoF carriers with *C9orf72* expansions should be interpreted in the context of oligogenic inheritance rather than as evidence of an isolated *NEK1* LoF-associated phenotype. Given that both *C9orf72* and *NEK1* are integral to DNA damage repair, their combined dysfunction likely converges to increase motor neuron vulnerability and drive disease progression. Indeed, iPSC models carrying both variants exhibit exacerbated DNA damage, suggesting that *NEK1* haploinsufficiency compounds the genomic instability induced by *C9orf72* expansions (Santangelo et al., 2024). This synergy may lower the cellular threshold for DNA damage tolerance, accelerating neurodegeneration through a combined molecular mechanism rather than a simple ‘second-hit’ effect. Consequently, *NEK1* variants may function as risk modifiers that contribute to clinical heterogeneity; however, the current data do not allow us to determine the independent phenotypic contribution of *NEK1* LoF variants, particularly in carriers with concomitant *C9orf72* expansions. This may also explain why the penetrance and clinical presentation of *NEK1* variants vary widely across cohorts. While our observations are consistent with *NEK1* acting as a risk modifier, the degree to which its effects are influenced by specific genetic or environmental factors remains to be elucidated through future controlled studies. Beyond motor phenotype, contemporary ALS research increasingly emphasizes the prognostic relevance of non-motor manifestations. In particular, recent classifications of mild behavioral and neurocognitive impairment in ALS indicate that subtle cognitive and behavioral changes, even in the absence of overt dementia, may carry important prognostic information (Spisto et al., 2025). In our cohort, no patient was documented as having overt dementia, but standardized ALS-specific cognitive and behavioral assessment was not systematically performed. Therefore, we were unable to distinguish patients with no cognitive-behavioral involvement from those with mild cognitive impairment, mild behavioral impairment, or combined mild cognitive-behavioral impairment, which limits the completeness of our phenotypic characterization. More importantly, this limitation reduces confidence in genotype–phenotype interpretation and means that the current data are insufficient to support fine-grained clinical subtyping of *NEK1*-associated ALS. Similarly, neuropathic pain in ALS should be interpreted in the context of emerging evidence that sensory involvement is not uncommon. Skin-biopsy studies have demonstrated degeneration of small sensory fibers across ALS clinical stages, supporting the view that at least a subset of patients exhibit peripheral sensory system involvement beyond the classical motor phenotype (Nolano et al., 2024). In our study, neuropathic pain was recorded descriptively, but no dedicated sensory work-up, including skin biopsy or quantitative sensory testing, was performed. As a result, we could not determine whether pain symptoms reflected small-fiber pathology or other nonspecific disease-related factors. Autonomic dysfunction was also not systematically evaluated in our cohort. This is relevant because prospective longitudinal data indicate that autonomic symptom burden in ALS is associated with faster disease progression and shorter survival (Dubbioso et al., 2023). Therefore, the absence of standardized autonomic assessment in our study may have led to under-recognition of clinically meaningful non-motor features with potential prognostic implications.

Our exploratory gene-based burden analysis suggested a possible enrichment signal of rare *NEK1* variants in our cohort compared with ancestry-matched gnomAD EAS controls. However, this comparison relied on an external summary-level reference dataset and could not account for individual-level ancestry principal components, sequencing batch, capture design, or platform-specific coverage differences. Therefore, this result should be interpreted as descriptive and supportive only, rather than as robustly adjusted evidence of genetic effect size. In particular, although restricting the comparison to the East Asian subset of gnomAD reduced broad ancestry mismatch, residual population stratification within East Asian subgroups could not be excluded. Moreover, differences between our Illumina NovaSeq 6,000 exome data and the heterogeneous sequencing architectures represented in gnomAD may have influenced variant detectability, especially for low-frequency or poorly covered sites. For this reason, the burden analysis is best viewed as a hypothesis-supporting sensitivity analysis. Our findings reinforce *NEK1*’s role as a risk modifier rather than a primary driver. The observed phenotypic variability likely stems from an interplay between the genetic background—such as *C9orf72* co-mutations—and systemic factors. The absence of direct *in vitro* functional or splicing assays further limits the definitive classification of rare variants; for instance, re-evaluating c.396+158A>G using current gnomAD data led to its reclassification as Likely Benign, highlighting the risk of incidental rare variants. While the extreme variability in sampling time, the use of peripheral leukocytes, and the lack of control for major clinical confounders substantially limit the interpretability of our protein analysis, these exploratory observations provide only a preliminary systemic perspective that warrants further validation in standardized, larger multi-center cohorts using longitudinal sampling.

To date, LoF variants have generally been considered more strongly associated with ALS risk than missense variants (Pensato et al., 2025; Brenner et al., 2016). However, in our cohort, caution is required when interpreting LoF-associated clinical features because 2 of the 3 LoF carriers also harbored pathogenic *C9orf72* repeat expansions. In our small cohort, carriers of different variant types had broadly similar ages at onset; however, any apparent differences in progression or survival should be regarded as preliminary descriptive observations and not as evidence for a distinct clinical phenotype attributable specifically to *NEK1* LoF variants. Such as *SETX*, fALS and sALS often differ markedly in age at onset, with fALS typically presenting much earlier and sALS clustering in middle to late adulthood (Chen et al., 2025). Only a small proportion of patients develop symptoms earlier, around the age of 40 years old (Jiang et al., 2023; Pensato et al., 2025; Riva et al., 2022; Shu et al., 2018). The rare juvenile ALS reported in the literature often also carry pathogenic variants in genes more strongly linked to early onset ALS (Sánchez-Tejerina et al., 2022). Although a nominal difference in age at onset was observed between Asians and Caucasians, the mean age at onset in both populations still fell within the 50–60-years range; therefore, this finding should be interpreted cautiously as a descriptive population-level trend rather than a definitive ethnic effect. In addition, our exploratory analysis of peripheral leukocyte *NEK1* levels suggested that lower measured protein abundance may be associated with faster clinical progression. However, because the samples were collected at highly heterogeneous disease stages, were derived from peripheral leukocytes rather than disease-relevant tissue, and were not adjusted for key confounders such as treatment exposure, nutritional status, respiratory status, and disease severity at the time of sampling, this observation should be regarded only as a preliminary hypothesis-generating signal and not as evidence of biomarker utility. Future studies would also be strengthened by integrating formal electrophysiological characterization, including EMG-based lower motor neuron assessment and TMS-based measures of cortical excitability, because these approaches may improve phenotypic stratification and provide additional prognostic information in ALS (Ranieri et al., 2025). In particular, EMG may help define the distribution, burden, and subclinical spread of lower motor neuron involvement more precisely, thereby improving discrimination between clinically overlapping motor phenotypes. TMS may capture corticospinal dysfunction and cortical hyperexcitability that are not evident from routine clinical examination alone, and these measures may be especially useful for identifying patients with more aggressive upper motor neuron system involvement. Together, EMG and TMS could improve interpretation of disease trajectory, support more refined phenotypic stratification, and enhance prognostic assessment in *NEK1*-associated ALS. What’s more, we further found that Asians more often present with upper limb onset and typically show the classic ALS phenotype, but in Europeans, flail arms and flail legs are reported more frequently, and the proportions of patients with a family history are also higher. Notably, even within a single cohort, marked clinical heterogeneity can be seen. Patients carrying the same variant may differ in site of onset, rate of progression, and survival. Its effects appear to depend more on coexisting variants, environmental exposures, and individual level biological differences, resulting in substantial variability in clinical phenotype and disease course.

This study has several limitations that warrant consideration, including the small number of *NEK1* variant carriers (*n* = 8) in our single-center cohort, which limits statistical power and generalizability, particularly for subgroup comparisons between LoF and missense variants; moreover, 2 of the 3 LoF carriers also harbored *C9orf72* repeat expansions, making it difficult to disentangle the independent clinical contribution of *NEK1* LoF variants. The cross-sectional design and variable sampling intervals for protein analysis (3–90 months post-onset) preclude definitive conclusions about temporal relationships. In the literature component, although the search was performed systematically, the review should be regarded as a structured narrative synthesis rather than a formal systematic review, because quantitative pooling was not performed. To improve transparency, we have now added a study selection flow diagram, explicit record counts for each screening stage, and a summary table of included studies; however, the review still does not aim to provide a PRISMA-based meta-analytic synthesis. In addition, co-mutation data—particularly *C9orf72* status—were incompletely reported in many published studies, which limits our ability to estimate the true prevalence and phenotypic effect of concomitant *C9orf72* expansions. For the rare-variant burden analysis, the use of gnomAD EAS as an external reference reduced broad ancestry mismatch but did not permit individual-level adjustment for population structure, sequencing batch, or platform-related coverage differences, which may have affected the precision of the reported odds ratios. Other limitations include protein quantification performed on peripheral blood leukocytes rather than disease-relevant tissues; substantial heterogeneity in sampling time (3–90 months after onset); lack of adjustment for potentially important confounders such as disease stage, treatment exposure, nutritional status, and respiratory function; lack of transcript-level validation for splice variants; absence of direct functional validation for VUS; incomplete segregation analysis because family DNA samples were unavailable for most cases; and the absence of systematic non-motor and neurophysiological phenotyping. In particular, standardized ALS-specific cognitive/behavioral classification, formal autonomic assessment, sensory evaluation for small-fiber involvement, detailed EMG characterization, explicit upper-versus-lower motor neuron burden assessment, and TMS-based cortical excitability measures were not available. Their absence is important because EMG could have improved characterization of lower motor neuron distribution and burden, whereas TMS could have provided complementary information on corticospinal dysfunction and cortical hyperexcitability. In a genetically heterogeneous cohort such as this one, these measures might have improved phenotypic stratification and strengthened prognostic interpretation by identifying clinically relevant differences not captured by routine examination alone. These missing measures limit our ability to define the full multisystem phenotype of *NEK1*-associated ALS and may also have affected prognostic interpretation, because cognitive-behavioral impairment, autonomic dysfunction, and cortical excitability abnormalities are increasingly recognized as clinically relevant outcome modifiers in ALS (Spisto et al., 2025; Dubbioso et al., 2023; Ranieri et al., 2025). The absence of EMG- and TMS-based metrics also limits our ability to determine whether electrophysiological differences parallel the observed genetic and clinical heterogeneity. Therefore, the pathogenic contribution of several non-LoF variants in this cohort remains uncertain. In our review of previously reported *NEK1*-ALS cases, 18 cases were explicitly reported to harbor concomitant *C9orf72* repeat expansions among 74 cases with available co-mutation information. However, because *C9orf72* screening was not consistently reported across all included studies, and many reports lacked complete variant-level co-mutation data, the true frequency of *C9orf72* co-occurrence is likely underestimated. This limitation should be considered when interpreting genotype–phenotype associations attributed to *NEK1* alone.

In summary, this study provides a descriptive overview of the *NEK1* genetic landscape in a Chinese ALS cohort and integrates these observations with a structured narrative review of previously reported cases. Rare *NEK1* variants were identified in approximately 2% of this cohort; however, the level of evidence differed across individual variants, and only the LP variants currently have stronger support for disease relevance. We also observed reduced peripheral *NEK1* protein levels among variant carriers in this small exploratory sample. Because these measurements were obtained from peripheral leukocytes, at highly heterogeneous sampling times, and without adjustment for major clinical confounders, they should be interpreted cautiously as hypothesis-generating observations rather than biomarker evidence. The observed inverse relationship between lower measured protein levels and faster clinical decline may help motivate future studies, but it does not establish biomarker utility or a direct mechanistic link. In addition, our literature synthesis highlights potential population differences in variant distribution and phenotype. Overall, these findings underscore the need for larger, multi-ethnic, prospectively phenotyped studies using standardized protocols.

Collectively, these descriptive insights expand the known *NEK1*-ALS spectrum. They also emphasize the importance of standardized, multi-ethnic longitudinal research to determine whether systemic protein dynamics are reproducible and biologically informative, while recognizing that the current data are not sufficient to support clinical biomarker claims.

## Data Availability

The raw data supporting the conclusions of this article will be made available by the authors, without undue reservation.
