# Supplementary material for: NEK1 variants and reduced protein levels in Chinese ALS patients: a descriptive study
Source: Front Aging Neurosci. 2026 May 21;18:1831861. doi: 10.3389/fnagi.2026.1831861 (PMC13233418; doi:10.3389/fnagi.2026.1831861)
Supplement: Supplementary file 1 [file Supplementary_file_1.docx]

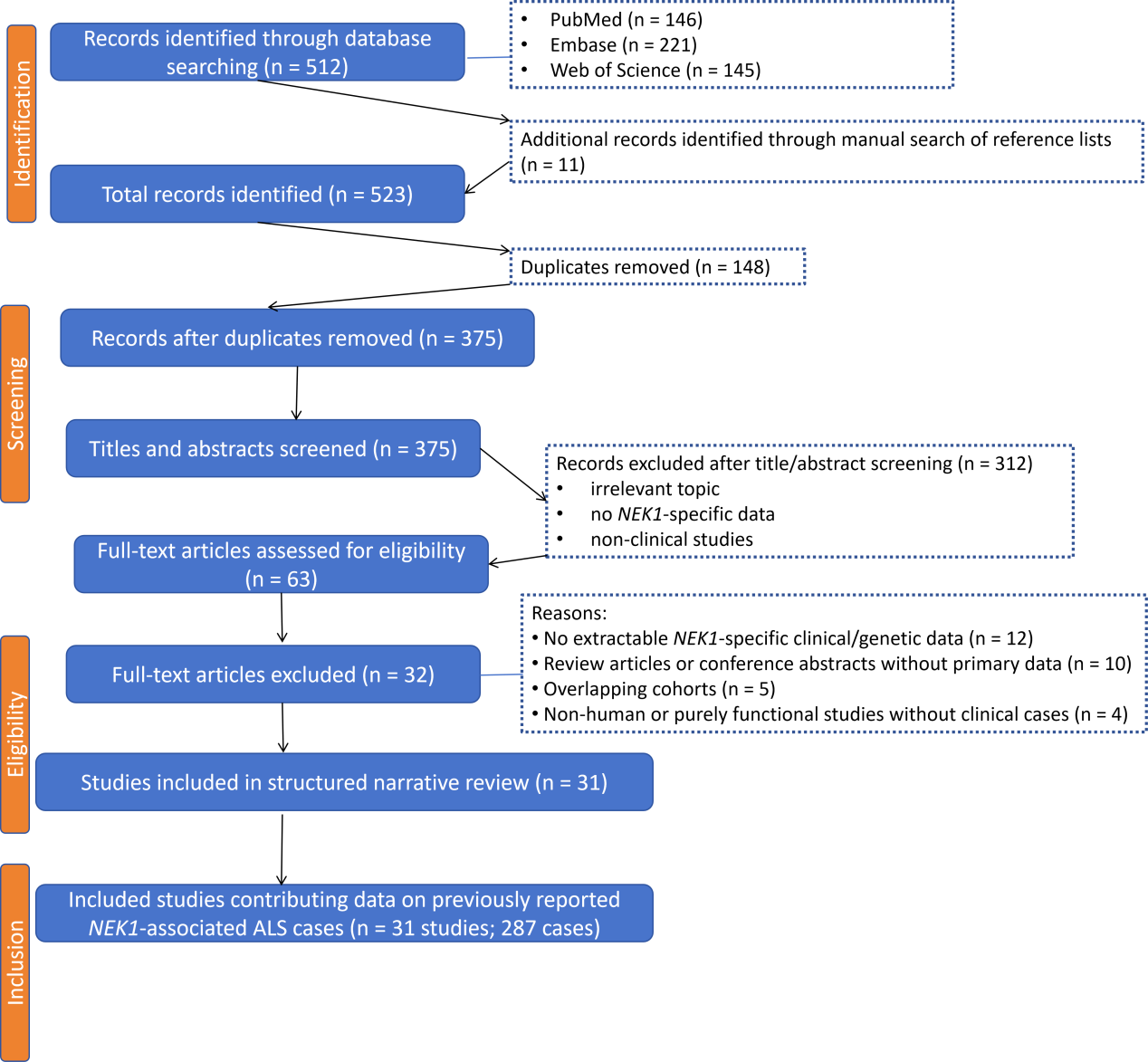


Supplementary Figure S1. Flow diagram of study selection for the structured narrative review of *NEK1*-associated ALS literature.


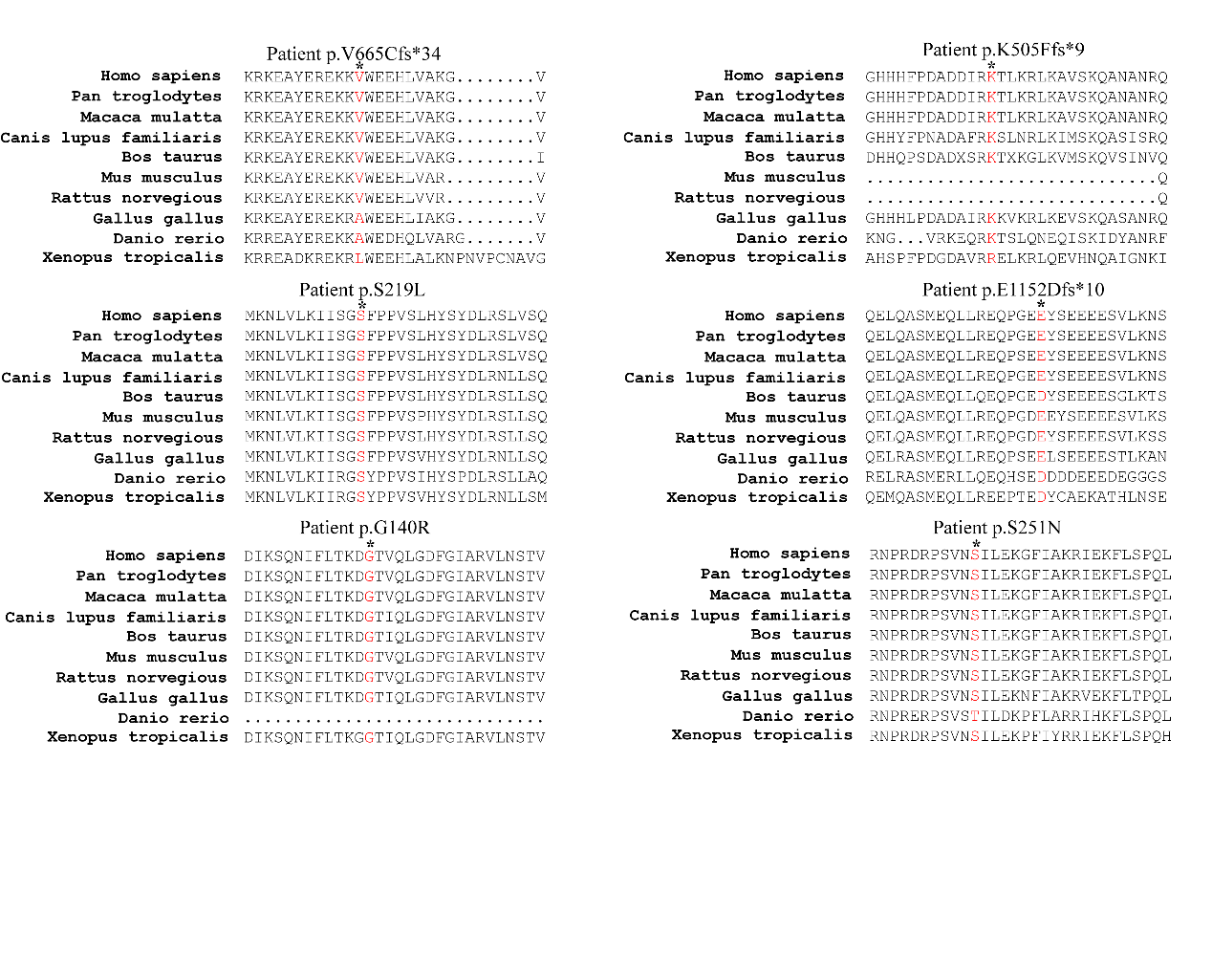


**Supplementary Figure 2** Imagine of species conservation analysis of the mutation sites in ALS patients with *NEK1* variants.

Supplementary Table S1. Studies related to NEK1-associated ALS included in the structured narrative review

| **First author, year** | **Ref. no.** | **Country / region** | **Study design** | **NEK1-associated ALS cases included*** | **Main NEK1 variant category reported** | **Age at onset available** | **Site of onset available** | **Survival available** | **Cognitive / behavioral data available** | **Co-mutation data available** | **Main note / contribution** |
| --- | --- | --- | --- | --- | --- | --- | --- | --- | --- | --- | --- |
| Gratten, 2017 | [9] | China | Case-control / exome study | NR | LoF + missense | Limited | No | No | No | No | Supported NEK1 as a risk gene in Chinese ALS |
| Naruse, 2021 | [15] | Japan | Sporadic ALS cohort | NR | LoF-dominant | Limited | Limited | No | No | No | Demonstrated increased risk of sporadic ALS with LoF variants |
| Tsai, 2020 | [23] | Taiwan, China | Cohort / phenotype study | NR | LoF-dominant | Yes | Yes | Limited | No | Limited | Highlighted hand-onset weakness as a common feature in NEK1 LoF carriers |
| Jiang, 2023 | [26] | China | Clinical-genetic cohort study | NR | LoF + missense | Yes | Yes | Yes | Limited | Yes | Main phenotype-oriented NEK1 ALS report |
| Pensato, 2025 | [27] | Italy | ALS genetic variability cohort | NR | Mixed | Yes | Yes | Limited | Limited | Yes | Recent Italian NEK1 cohort with clinical variability |
| Sánchez-Tejerina, 2022 | [28] | Spain | Case report / literature review | 1 | Mixed / co-mutated context | Yes | Yes | Limited | No | Yes | Included atypical juvenile-onset ALS in an oligogenic context |
| Riva, 2022 | [29] | Italy | ALS cohort study | NR | Mixed | Yes | Yes | Limited | No | Yes | Reported clinical and genetic heterogeneity of NEK1 carriers |
| Ma, 2022 | [30] | China | Familial / young-onset ALS cohort | NR | Mixed | Yes | Limited | No | No | Limited | Included Chinese familial or young-onset cases |
| Forsberg, 2019 | [31] | Europe | Neuropathology / genetics study | NR | Mixed | No | No | No | No | Yes | Included patients with variants in C9orf72 and other ALS genes |
| Black, 2017 | [32] | Scotland | Population genetic epidemiology study | NR | Mixed | No | No | No | No | Limited | Population-level frequency data |
| Yilmaz, 2022 | [33] | Germany | Case report | 1 | Mixed | Yes | Yes | No | No | Yes | Example of highly oligogenic sporadic ALS |
| Shu, 2018 | [34] | China | Mutation screening study | NR | Mixed | Limited | Limited | No | No | No | Chinese screening cohort focused on NEK1 variants |
| Nel, 2022 | [35] | Southern Africa | Cohort study | NR | Mixed | Limited | Limited | No | No | Limited | Expanded mutational spectrum outside Europe/Asia |
| McCann, 2020 | [36] | Australia | Sporadic ALS genetic study | NR | Mixed | Limited | No | No | No | Yes | Supported polygenic / oligogenic contribution |
| Müller, 2018 | [37] | Germany | Familial ALS cohort | NR | Mixed | Limited | Limited | Limited | No | Limited | Comprehensive analysis of mutation spectrum in German ALS families |
| Chen, 2022 | [38] | Mainland China | Large cohort study | NR | Mixed | Limited | Limited | Limited | No | Limited | Large Chinese mainland ALS genetics cohort |
| Brenner, 2016 | [39] | Germany | Familial ALS cohort | NR | Mainly LoF | Limited | Limited | Limited | No | Limited | Early report emphasizing NEK1 mutations in familial ALS |
| Lattante, 2021 | [40] | Italy | Case series + cellular study | NR | Missense-focused | Limited | Limited | No | Limited | Limited | Functional support for some missense variants |
| Tripolszki, 2019 | [41] | Hungary | Cohort study | NR | Mixed | Limited | Limited | No | No | Limited | Comprehensive genetic analysis of a Hungarian ALS cohort |
| Liu, 2019 | [42] | China | Cohort study | NR | Mixed | Limited | Limited | No | No | Limited | Genetic spectrum and variability in Chinese ALS patients |
| Liu, 2021 | [43] | Central South China | Cohort study | NR | Mixed | Limited | Limited | No | No | Limited | Mutation spectrum study of ALS in Central South China |
| Nguyen, 2018 | [44] | Belgium | Cohort study (ALS and ALS-FTD) | NR | Mixed | Limited | Limited | No | Limited | Limited | NEK1 genetic variability in a Belgian cohort of ALS and ALS-FTD patients |
| Zhang, 2020 | [45] | China (Hui descent) | Case report | 1 | Mixed / co-mutation context | Yes | Yes | Limited | No | Yes | Reported coexistence of NEK1 and GRN mutations in a sporadic ALS patient |
| Bartoletti-Stella, 2021 | [46] | Italy | Targeted-panel cohort study | NR | Mixed | Limited | Limited | No | Limited | Limited | Targeted sequencing in Italian ALS patients supporting etiologic heterogeneity across the ALS/FTD continuum |
| Libonati, 2024 | [47] | Italy | Cohort study | NR | Mixed | Limited | Limited | No | No | Limited | Italian ALS genetics-screening cohort emphasizing early testing |
| Olsen, 2024 | [48] | Multinational / Scandinavian | Cohort study | NR | Mixed | Limited | Limited | No | No | Limited | Reported genetic overlap between ALS and other neurodegenerative / neuromuscular disorders |
| Leighton, 2024 | [49] | Scotland | Cohort study | NR | Mixed | Yes | Yes | Limited | Limited | Limited | Updated Scottish genotype–phenotype landscape in motor neuron disease |
| Santangelo, 2024 | [50] | Italy | Experimental study with patient-derived iPSC material | NR | NEK1 haploinsufficiency / LoF context | No | No | No | No | Yes | Included by reference number in current review set; primarily mechanistic study in C9ORF72 patient-derived iPSC motoneurons |
| Rifai, 2025 | [51] | Multicenter / Europe-dominant | Clinicopathological analysis | NR | Mixed | Limited | Limited | Limited | Limited | Limited | Clinicopathological analysis of NEK1 variants in ALS |
| Ramos, 2019 | [52] | Greece | Genetic screening cohort | NR | Mixed | Limited | Limited | No | Limited | Limited | Included by reference number in current review set; title indicates FTD-spectrum genetic screening rather than ALS-specific NEK1 cohort |
| Yang, 2025 | [53] | China | Case report / literature review | 1 | LoF (frameshift) | Yes | Yes | Limited | No | Limited | Novel NEK1 frameshift ALS case with literature review |

Abbreviations: ALS, amyotrophic lateral sclerosis; LoF, loss of function; NR, not reported or not extractable from the current manuscript summary.

* “NEK1-associated ALS cases included” refers to the number of ALS patients carrying NEK1 variants reported in each referenced study when extractable from the current manuscript text.

Note: This version is constructed strictly according to the reference numbers specified in the current manuscript: [9], [15], [23], [26]–[53]. Where the present manuscript does not provide study-level details, fields are marked as NR or Limited rather than being inferred beyond the available text. References [47]–[53] were checked against the manuscript reference list; refs [50] and [52] are retained here only because they fall within the user-specified reference range, even though their titles suggest they may not fit the same clinical-review scope as the core NEK1-ALS cohort studies.

**Supplementary Table 2.** Exploratory Descriptive Comparison of Rare NEK1 Variants in the ALS Cohort and the gnomAD EAS Reference Dataset

| **Variant category** | **Frequency criterion in gnomAD EAS** | **Frequency in ALS cohort** | **Frequency in gnomAD EAS reference** | **Nominal p-value*** | **Exploratory OR*** | **95% CI*** |
| --- | --- | --- | --- | --- | --- | --- |
| NEK1 rare variants (total) | <0.1% | 1.85% (7/378) | 0.58% | 0.003 | 3.25 | 1.51–6.98 |
| Frameshift deletions | <0.1% | 0.79% (3/378) | — | 0.002 | 3.6 | 1.25–9.15 |
| Splice variants | <0.1% | 0.53% (2/378) | — | 0.005 | 4.2 | 1.58–11.21 |
| Missense variants | <0.1% | 0.53% (2/378) | — | 0.02 | 2.8 | 1.11–7.06 |
| Excluded variant (c.396+158A>G) | >0.1% | — | — | — | — | — |

* Exploratory descriptive estimates derived from comparison with an external summary-level reference dataset.

**Note:** This table is provided for **descriptive transparency only.** The comparison was restricted to rare NEK1 variants (MAF < 0.1%) in coding or canonical splice-related regions captured by the case exome panel. Case samples were sequenced on the Illumina NovaSeq 6000 platform with mean coverage >100× and ≥95% of target bases covered at ≥20×, whereas gnomAD EAS represents an **external summary-level reference dataset** aggregated across multiple sequencing platforms and pipelines. No individual-level correction for ancestry principal components, sequencing batch, platform-specific coverage, or other potential confounders was possible. Accordingly, the reported odds ratios, confidence intervals, and nominal p-values should be interpreted as **exploratory descriptive estimates**, rather than as definitive measures of genetic effect size or formal association.
